# Supplementary material for: Using dried blood spots to estimate Toxoplasma gondii seroprevalence in pregnant women in Catalonia, Spain, and to serologically diagnose congenital toxoplasmosis
Source: PLoS Negl Trop Dis. 2026 Jan 5;20(1):e0013881. doi: 10.1371/journal.pntd.0013881 (PMC12782418; doi:10.1371/journal.pntd.0013881)
Supplement: S1 Table — (DOCX) [file pntd.0013881.s001.docx]

**S1 Table. *Toxoplasma gondii* IgM assay validation**

|  | **Summary of IgM assay variation during the initial method verification** | | | | | | | | |  |  |
| --- | --- | --- | --- | --- | --- | --- | --- | --- | --- | --- | --- |
|  |  | | SAMPLES | | | | | | |  |  |
|  |  | | n=10 | | **S1** | **S2** | |  | |  |  |
|  | **Mean (ratio)** | |  | | 0.16 | 3.49 | |  | |  |  |
|  | **SD (ratio)** | |  | | 0.04 | 0.62 | |  | |  |  |
|  | **kit SD expected values** | |  | | <0.53 | <0.88 | |  | |  |  |
|  | **CV (%)** | |  | | 25.1 | 17.7 | |  | |  |  |
|  | **kit CV expected values** | |  | | <27 | <27 | |  | |  |  |
|  |  | |  | |  |  | |  | |  |  |
|  |  | | n | | **Value** |  | |  | |  |  |
|  |  | |  | |  |  | |  | |  |  |
|  | **External Quality assessment** | | 5 | | 100% |  | |  | |  |  |
|  |  | |  | |  |  | |  | |  |  |
|  | **External Quality inter-lab assessment** | | 11 | | 100% |  | |  | |  |  |
|  |  | |  | |  |  | |  | |  |  |
| n=5 | | | | | **CDC samples** | | | | | | |
|  |  |  |  |  | positive | | | doubtful | | negative | |
| **Euroimmun ELISA anti *Toxoplasma gondii* (IgM)** | | | positive | | 3 | | | 0 | | 0 | |
|  |  |  | doubtful | | 0 | | | 0 | | 0 | |
|  |  |  | negative | | 0 | | | 0 | | 2 | |

| n=11 | | **Serum ELISA method** | | |
| --- | --- | --- | --- | --- |
|  |  | positive | doubtful | negative |
| **Euroimmun ELISA anti *Toxoplasma gondii* (IgM)** | positive | 7 | 0 | 0 |
|  | doubtful | 1 | 0 | 0 |
|  | negative | 0 | 0 | 3 |

*Abbreviations: CT, congenital toxoplasmosis; CV, coefficient of variation (%); DBS, dry blood spot; NB, newborns; SD, standard deviation (dimensionless).*

- Validation was performed considering the interpretation of results as a qualitative mode. To this end, the study was conducted by calculating the ratio between the absorbance of the sample and that of Stardard 2 provieded by kit supplier’s. Ratios are dimensionless.
- Sample 1 (S1, negative quality control) and Sampe 2 (S2, positive quality control) were from lot E231222AD.
- Variation was expressed as standard deviations (SD) and CV%, both were compared with the kit supplier’s SD and CV% for the same range of values.
- External quality assessment (DBS samples) was evaluated as a qualitative method (CDC Program, % of successful results are indicated; also number of concondant results are indicated). Sensitivity and Specificity was 100%.
- External quality inter-laboratory assessment was evaluated as a qualitative method (serum samples from NB with confirmed Toxoplasma-IgM results, number of concordant results are indicated). Sensitivity and Specificity were 87,5% and 100%, repectively.
