# Supplementary material for: Using dried blood spots to estimate Toxoplasma gondii seroprevalence in pregnant women in Catalonia, Spain, and to serologically diagnose congenital toxoplasmosis
Source: PLoS Negl Trop Dis. 2026 Jan 5;20(1):e0013881. doi: 10.1371/journal.pntd.0013881 (PMC12782418; doi:10.1371/journal.pntd.0013881)
Supplement: S2 Table — (DOCX) [file pntd.0013881.s002.docx]

**S2 Table: *Toxoplasma gondii* IgG assay validation.**

|  | **Summary of IgG assay variation during the initial method verification** | | | | |  |
| --- | --- | --- | --- | --- | --- | --- |
|  |  | SAMPLES | | | |  |
|  |  | N=35 | **S1** | **S2** | **S3** |  |
|  | **Mean (ratio)** |  | 0.25 | 2.72 | 4.46 |  |
|  | **SD (ratio)** |  | 0.03 | 0.24 | 0,54 |  |
|  | **kit SD expected values** |  | <0.29 | <0.47 | <0.57 |  |
|  | **CV (%)** |  | 10.3 | 8.9 | 12.2 |  |
|  | **kit CV expected values** |  | <14 | <14 | <14 |  |

|  |  | | | n | | **Value** | |  | |  |  |  |
| --- | --- | --- | --- | --- | --- | --- | --- | --- | --- | --- | --- | --- |
|  |  | | |  | |  | |  | |  |  |  |
|  | **External Quality inter-lab assessment** | | | 26 | | 100% | |  | |  |  |  |
|  |  | | |  | |  | |  | |  |  |  |
|  | | |  |  | |  | |  | |  | |  |
|  | | | n=26 | | | **Serum ELISA method** | | | | | |  |
|  | | |  |  |  | positive | | doubtful | | negative | |  |
|  | | | **Euroimmun ELISA anti Toxoplasma gondii (IgG)** | positive | | 23 | | 0 | | 0 | |  |
|  | | |  | doubtful | | 0 | | 0 | | 0 | |  |
|  | | |  | negative | | 0 | | 0 | | 3 | |  |

*Abbreviations: CT, congenital toxoplasmosis; CV, coefficient of variation (%); DBS, dry blood spot; NB, newborns; SD, standard deviation (dimensionless).*

- Validation was performed considering the interpretation of results in semi-quantitative mode. To this end, the study was conducted by calculating the ratio between the absorbance of the sample and that of Stardard 2 provieded by kit supplier’s. Ratios are dimensionless.
- Sample 1 (S1, negative quality control), Sampe 2 (S2, positive quality control) and Sample 3 (S3, Standard-S1) were from lot E240322AV.
- Variation was expressed as standard deviations (SD) and CV %, both were compared with the kit supplier’s SD and CV % for the same range of values.
- External quality inter-laboratory assessment was evaluated as a qualitative method (DBS samples from NB with confirmed Toxoplasma-IgG positive mothers, % of successful results are indicated).
- External quality inter-laboratory assessment was evaluated as a qualitative method (serum samples from NB with confirmed Toxoplasma-IgG results, number of concordant results are indicated). Both Sensitivity and Specificity were 100%.
