# Supplementary material for: Using dried blood spots to estimate Toxoplasma gondii seroprevalence in pregnant women in Catalonia, Spain, and to serologically diagnose congenital toxoplasmosis
Source: PLoS Negl Trop Dis. 2026 Jan 5;20(1):e0013881. doi: 10.1371/journal.pntd.0013881 (PMC12782418; doi:10.1371/journal.pntd.0013881)
Supplement: S3 Table — (DOCX) [file pntd.0013881.s003.docx]

**S3 Table: Demographic data from Catalonian newborns’ mothers.**

| **Pregnant women by age (years)** | | |
| --- | --- | --- |
| n (newbons) | **3200*** | **98857** |
| **<25 years** | 271 (8,5%) | 9320 (9,4%) |
| **26-35 years** | 1598 (49,9%) | 52681 (53,3%) |
| **>36 years** | 1297 (40,5%) | 35922 (36,3%) |
| **Unknown** | 34 (1,1%) | 934 (0,9%) |

| **Pregnant women by origin** | | |
| --- | --- | --- |
| N (newborns) | **3200*** | **98857** |
| **Spanish** | 2067 (64,6%) | 60618 (61,3%) |
| **Foreign** | 1102 (34,4%) | 36829 (37,3%) |
| **Unknown** | 31 (1%) | 1410 (1,4%) |

*Demographic values ​​were similar to those observed in the general population, which were calculated based on the corresponding number of live births (n=98857) in Catalonia during the study period according to official records from our NBS Program. *Samples included in our seroprevalence study.*
