# Supplementary material for: Using dried blood spots to estimate Toxoplasma gondii seroprevalence in pregnant women in Catalonia, Spain, and to serologically diagnose congenital toxoplasmosis
Source: PLoS Negl Trop Dis. 2026 Jan 5;20(1):e0013881. doi: 10.1371/journal.pntd.0013881 (PMC12782418; doi:10.1371/journal.pntd.0013881)
Supplement: Attachment — Submitted filename: RESPONSE TO REVIEWERS.docx [file pntd.0013881.s006.docx]

**RESPONSE TO REVISORS**

**Dear Editors and Reviewers,**

Thank you very much for the time and effort you invested in reviewing our manuscript *“Using dried blood spots to estimate Toxoplasma gondii seroprevalence in pregnant women in Catalonia (Spain), and to serologically diagnose congenital toxoplasmosis”.* We appreciate the valuable feedback and constructive comments provided, which have helped us improve the quality and clarity of our work.

Below, we provide a detailed point-by-point response to each comment, describing the changes made in the revised manuscript. All modifications have been highlighted in red in the revised version of the manuscript.

**1. Title and Abstract**

**Reviewer #2 comment:** *Shorten the title and include ELISA test type in abstract.*

**Response:**
We have shortened the title to: *“Using dried blood spots to estimate Toxoplasma gondii seroprevalence in pregnant women in Catalonia (Spain) and to serologically diagnose congenital toxoplasmosis”*.
Additionally, we have included the serological method used (ELISA) in the abstract (Line 54).

**2. Introduction**

**Reviewer #3 comment:** *Add a brief explanation of the differences between IgG and IgM antibodies.*

**Response:**
We added a paragraph in the Introduction (Lines 122-134) explaining the biological differences and diagnostic implications of IgG versus IgM antibodies to improve reader understanding.

**Reviewer #4 comment:** *no explicit hypothesis is stated.*

**Response:**

We appreciate the reviewer’s comment. Indeed, we did not state an explicit hypothesis in the original version. In the revised manuscript, we have now included it at the end of the Introduction section (Lines 161-162).

**3. Methods**

**Reviewer #1, #2, #4 comments:**

- *Clarify sample selection and population definitions.*
- *Validation of DBS methodology*
- *Add details on DBS sample storage and handling.*
- *Sample size calculation for Aim 2*
- *Provide ethical approval information.*
- *Add statistical methods with confidence intervals and regression analyses.*

**Response:**

- A flow diagram (Figure 2, A and B) illustrates the selection process for CT cases. From 3,231 newborn samples, 3,200 maternal DBS were included after excluding twins. Similarly, from 63 initial CT cases, 30 met inclusion criteria and had available DBS for analysis.
- Thank you for your insightful comment regarding the validation of our method using dried blood spots (DBS). We would like to clarify that this validation has already been conducted and is included in our study. The results demonstrate that our method (IVD) performs reliably with DBS samples, showing comparable accuracy and sensitivity to standard protocols. For further details, please refer to S1 and S2 Tables where validation data are shown.
- Details on DBS storage conditions, handling, and processing are now included in the Methods section (Lines 229-231) to address concerns about antibody stability, especially for IgM detection.
- We appreciate the reviewer’s comment. However, no sample size calculation was performed for Objective 2, as this analysis was not part of the main study objective. The evaluation of DBS for IgM detection in congenital toxoplasmosis was conceived as an exploratory subanalysis of positive IgM samples, aimed at assessing their potential usefulness for retrospective diagnosis or neonatal screening of toxoplasmosis.
- Ethical approval was obtained from Trueta Ethics Committee (approval number: CEIm code 2018.027) and Carlos III Health Research Institute Ethics Committee (CEIm code PI42_2022) and by the local ethics committee of each participating center, as detailed on Lines 246-253.
- We expanded the statistical analysis section to include confidence intervals for sensitivity, specificity (Lines 264-265 and 325-326)
- We agree with the reviewer that a univariate and multivariate analysis would have been useful to explore potential predictors of DBS positivity. However, due to limited sample size and the very low number of positive DBS samples (only one case), such analysis were not statistically feasible. Therefore only descriptive analyses were performed, as stated in the revised manuscript (Limitations section: Lines 486-489)

**4. Results**

**Reviewer #2, #4 comments:**

- Reconcile denominators and clarify exclusions in Table 1.
- Present full cohort characteristics including maternal infection trimester and clinical data.
- Include confidence intervals for sensitivity and specificity of IgM detection.
- Report results for CDC control samples and serum IgG data.

**Response:**

- We updated Table 1 and the corresponding text to ensure denominators are consistent and all exclusions are explained (Lines 283-284 and 291 and Figure2A).
- A new table (Table 2) presents detailed clinical and demographic characteristics of the CT cohort, including infection trimester, prenatal treatment, and neonatal clinical presentation (Lines 331-333).
- Sensitivity and specificity values for IgM detection now include 95% confidence intervals (Lines 325-326).
- Results for the 5 CDC control DBS samples as supplementary material (S1 and S2 Table), confirming the performance of the ELISA kit in these controls.
- IgG detection in serum samples from CT cases was also evaluated and the data are presented in the Results section (Table 2).

**5. Figures and Tables**

**Reviewer #1, #2, #4 #5 comments:**

- Improve Figure 1 resolution and format.
- Add PRISMA-style flow diagrams for sample selection.
- The relevance of Figure 1 for this article has been questioned

**Response:**

- Figure 1 has been revised and uploaded to meet PLOS requirements, with a resolution of at least 300 dpi and vector format.
- We added two flow diagrams summarizing sample selection for both seroprevalence and CT cohorts (Figures 2A and B), clarifying reasons for exclusion and final sample sizes.
- We thank the reviewer for this thoughtful comment. We respectfully consider that Figure 1 is relevant to the manuscript, as it provides a clear and structured overview of the different levels or strategies for the prevention of congenital toxoplasmosis. This figure is intended to facilitate the reader’s understanding of the preventive framework in which the study is contextualized. In addition, most of these strategies have been evaluated in studies conducted by the REIV-TOXO group, further supporting the appropriateness of including this figure in the main text.

**6. Discussion and Conclusions**

**Reviewer #4 comments:**

- Moderate claims about prenatal screening effectiveness.
- Acknowledge limitations including small CT sample size and antibody degradation in DBS.
- Link findings to public health implications.
- Suggest standardized protocols for the storage and handling of dried blood spot samples

**Response:**

- We tempered statements regarding the effectiveness of prenatal screening, noting that our study did not perform a direct comparison of screening strategies (Lines 495-499).
- The limitations section now explicitly discusses the small CT sample size, potential statistical imprecision, and challenges related to antibody stability in DBS (Lines 468-483). Additionally, the standardized conditions for storage and handling of samples are indicated and referenced (Lines 455-459)
- We expanded the public health discussion to highlight how our findings can inform screening policies and interventions targeting high-risk populations (Lines 501-505).
- We agree with the reviewers that establishing standardized protocols for the storage and handling of dried blood spot samples is essential. Such protocols would minimize pre-analytical variability that may compromise antibody detection. Based on our experience, we suggest storing samples at controlled room temperature in low-humidity conditions, protecting them from direct light, and transferring them to a −80 °C freezer for long-term storage. Elution procedures should be standardized, including the use of the same punch size, buffer composition, and incubation time. Implementing these standardized procedures would strengthen the reliability of future diagnostic and seroprevalence studies. This point has been added to the Discussion section (Lines 455–459).

**7. Editorial and Formatting Issues**

**Reviewer #1, #2 comments:**

- Correct funding statement formatting.
- Harmonize reference styles and journal abbreviations.
- Define all abbreviations at first use.
- Address minor typographical errors.
- Different denominators in Table 1.
- In the results you state that only 3152/3200 babies were born to residents of Catalonia. If your goal is to assess the seroprevalence of pregnant mothers in Catalonia specifically, should you not limit your population to residents and exclude mothers that reside outside of Catalonia?
- What was the odds ratio for pregnant women <25 years compared to pregnant women >35 years? Or compared to pregnant women 25-35 years? It might be interesting to look at trend across all three age categories.
- We noticed that you used the phrase 'data not shown' in the manuscript. We do not allow these references, as the PLOS data access policy requires that all data be either published with the manuscript or made available in a publicly accessible database. Please amend the supplementary material to include the referenced data or remove the references.

**Response:**

- The funding statement has been reformatted to include author initials, full grant numbers, and funder names per PLOS guidelines (Lines 550-554).
- Reference formatting has been standardized across the manuscript according to journal style.
- All abbreviations are now defined at first mention.
- Minor typographical and stylistic corrections have been applied throughout the manuscript, except for replacing *antenatal* with *prenatal*, because *antenatal* is the preferred term in European and international usage, including by the World Health Organization (WHO), and aligns with the overall style of the manuscript.
- We thank the reviewer for this observation. We have revised the text to clarify how the study population and denominators were defined. Denominators in Table 1 vary across variables because information on some demographic characteristics (e.g., mother’s origin, age, or place of residence) was missing in the NBS database for a small number of cases. These differences are now indicated in the Table 1 footnotes and are reflected in the manuscript (lines 283–291).
- We appreciate the reviewer’s comment. We retained the 48 non-resident mothers/newborns (48/3,200; 1.5%) in the primary analysis to preserve the representativeness of the Catalonia NBS screening program, which is defined by place of delivery rather than legal residence. Given their very small proportion, their inclusion is unlikely to materially affect the estimates. Moreover, residence recorded at delivery does not necessarily reflect postnatal residence; some of these infants may subsequently reside in Catalonia after birth.
- We examined maternal age categories (<25, 25–35, >35) and the corresponding odds ratios; the contrasts referenced by the reviewer were not statistically significant, and there was no evidence of a linear trend across categories (all p>0.05). Given the small number of outcomes in the <25 group, limited statistical power may have contributed to the absence of statistically significant differences. To avoid overinterpretation of non-significant findings and to keep the Results concise, we did not include these estimates in the main text. We would be pleased to provide them in the Supplementary Material at the Editor’s discretion.
- Thank you for your comment. Although demographic data were not included in the main manuscript, we have now added this information as supplementary material (S3 Table) to provide a more comprehensive overview of the study population. We believe this additional data will enhance the clarity and transparency of our work (Line 276).

**8. Reproducibility and Data Sharing**

Consider sharing an anonymized dataset (e.g., maternal age, origin, serological status) to ensure transparency and compliance with the open data policy.

**Response:**
There are ethical restrictions on sharing data set because it contains potentially

identifying and sensitive patient information and this restriction is imposed by the

Research Ethics Committee.

To request data please contact the Research Ethics Committee of Hospital Universitari

de Girona Dr. Josep Trueta ([ceic.girona.ics@gencat.cat](mailto:ceic.girona.ics@gencat.cat)) and Ethics Committee of Instituto de Salud Carlos III ([registro.general@isciii.es](mailto:registro.general@isciii.es)).

There is no third party organization that has access to the complete data included in

the article.

**Thank you again for your careful review and constructive suggestions. We believe these revisions have strengthened the manuscript substantially and look forward to your feedback.**

Sincerely,
Borja Guarch, on behalf of all authors
