# Supplementary material for: Using dried blood spots to estimate Toxoplasma gondii seroprevalence in pregnant women in Catalonia, Spain, and to serologically diagnose congenital toxoplasmosis
Source: PLoS Negl Trop Dis. 2026 Jan 5;20(1):e0013881. doi: 10.1371/journal.pntd.0013881 (PMC12782418; doi:10.1371/journal.pntd.0013881)
Supplement: Attachment — Submitted filename: Response to Reviewers II.docx [file pntd.0013881.s007.docx]

**RESPONSE TO REVISORS II**

**Dear Editors and Reviewers,**

Thank you very much for the time and effort you invested in reviewing our manuscript *“Using dried blood spots to estimate Toxoplasma gondii seroprevalence in pregnant women in Catalonia (Spain), and to serologically diagnose congenital toxoplasmosis”.*

We have carefully revised the manuscript according to their comments. Below, we provide detailed, point-by-point responses explaining the changes made and clarifications provided. All modifications are marked in the revised manuscript.

**1. Methods**

**-No explicit hypothesis statement (REVIEWER #1, #4)**

We have added a paragraph in the Introduction (Lines 163–165) including the formal, testable hypothesis statement as recommended by the reviewers.

**- Clarify the prospective vs. retrospective components in the Methods section (REVIEWER #1)**

Thank you for this comment. We have reviewed the text, and both the seroprevalence study (line 178) and the detection of *T. gondii*-specific IgM in confirmed CT cases (line 197) were already explicitly described as retrospective. We have carefully verified the manuscript to ensure that this information is clearly presented.

**-Clarity could be added on how “foreign-born” mothers were categorized (by birth country or nationality)**. **(REVIEWER #1)**
We have added this clarification in Methods, Study Population (line 188-189):

“Mothers were classified as foreign-born based on country of birth, regardless of current nationality.”

**-Specify how borderline results were treated statistically (they were reclassified as negative, but this should be justified). (REVIEWER #1)**

We thank the reviewer for this helpful comment. Borderline results were reclassified as negative for the statistical analyses. We adopted this conservative approach to avoid overestimating seroprevalence, considering only unequivocally positive results as positive. Although this may lead to a slight underestimation, we judged it preferable to the risk of inflating the number of true positives. This clarification has now been added to the Methods section (lines 247-249).

**- The retrospective design for CT case analysis limits conclusions about diagnostic sensitivity, as long storage time may have affected IgM stability The design does not allow a direct comparison with serum collected at birth weakening conclusions about neonatal screening feasibility (REVIEWER #4).**

We respectfully disagree with this comment. As detailed in the Results section (lines 327–329), IgM results from peripheral blood collected at birth were available for 27 of the 30 cases, allowing a direct comparison with IgM detection in DBS samples. The finding of IgM positivity in 10/27 serum samples at birth, contrasted with only 1/10 positive in DBS samples, indicates that IgM detection in both sample types is suboptimal and therefore unsuitable for neonatal screening.

The Methods section has now been updated to specify the retrospective nature of the CT case analysis and to acknowledge the potential impact of long-term storage on IgM stability (lines 234–235). This limitation is also addressed in the Discussion.

**- Representativeness of the 5.7% sample of total births was validated statistically? (REVIEWER #4)**

The sample size was calculated a priori, ensuring an adequate level of precision for estimating seroprevalence. Therefore, the 5.7% sample of total births is statistically appropriate and sufficiently representative for the purposes of this study.

 **- Statistical Analysis (REVIEWER #4): No adjustment for potential multiple testing or missing data imputation was described.**
**The authors did not include goodness-of-fit or model diagnostics for logistic regression. Sensitivity/specificity estimation for IgM was limited by small sample and retrospective bias.**

We thank the reviewer for the comment. Although these validation analyses were performed, we decided not to include them in the manuscript to avoid adding additional statistical detail that would complicate the presentation of results. For completeness, we summarise them here:

- **Goodness-of-fit (Hosmer–Lemeshow test):** *p* = 0.67726, indicating good model fit (*p* > 0.05; no significant difference between observed and predicted values).
- **Likelihood Ratio Test:** *p* < 0.001, showing that the included variables significantly improve prediction compared with the null model.
- **Predictive performance (AUC):**
  AUC = 0.6318 (95% CI 0.6049–0.6587), indicating a moderate ability of the model to discriminate between positive and negative subjects.
- **Missing data:** No imputation was performed because the proportion of missing values was very small and missing completely at random, thus not affecting the results.

**- Reliability of DBS testing. Where did the negative quality control come from? The sensitivity for IgG appears to be 100%, but how about the specificity? (REVIEWER #4)**

We have reviewed and extended the description of assay validation (Supporting information, S2 Table. *Toxoplasma gondii* IgG assay validation):

Negative quality control samples were derived from pre-screened, seronegative donors (serum samples from newborns with confirmed Toxoplasma-IgG results by ELISA). Specificity for IgG detection was estimated at 100%, consistent with the manufacturer’s data and our own validation using paired serum samples.

**- IgG IgG sensitivity could also be compromised by the same storage issues? Is the IgG sensitivity missed true positives, leading to an underestimation of the reported 15.5% seroprevalence? (REVIEWER #4)**

We have expanded the Discussion section (lines 466-471) to include this consideration:

“Although the low IgM sensitivity may partly reflect sample degradation, prolonged storage could theoretically also reduce IgG detectability, potentially leading to an underestimation of seroprevalence. Nevertheless, this scenario is unlikely, given the inherent stability of IgG, its higher concentration, and the fact that samples were stored for no longer than one year. Consequently, all these factors may explain the 10-fold higher detection of IgG compared to IgM, which ultimately affects their detectability [23,56]”.

**2. Results**

**-  Some numerical details are repeated both in text and tables (e.g., specific p-values and percentages). Summarize trends in the text and leave exact values in tables. (REVIEWER #4)**

Thank you for this observation. We have reviewed the manuscript and removed repeated numerical details from the text when they were already presented in the tables. The text now summarizes the main trends, while exact values (e.g., specific p-values and percentages) are reported exclusively in the tables, as recommended.

**- Some statistical details (e.g., exact p-values, CI bounds for odds ratios, and full logistic model parameters) are absent. There is no table showing multivariate regression output, only narrative interpretation. (REVIEWER #4)**

Thank you for this thoughtful comment. We have carefully considered your request; however, we believe that including the full multivariate analysis falls outside the primary objectives of our study. Moreover, adding all numerical parameters from the multivariate model would substantially increase the amount of statistical detail, potentially complicating the readability of the manuscript and distracting readers from the key findings we aim to highlight. For these reasons, we have chosen to maintain the current level of reporting. We believe that this approach preserves the clarity and focus of the manuscript while remaining aligned with the study’s objectives.

- **There is no explicit comparison of planned versus executed analyses for the CT group. (REVIEWER #4)**

Thank you for this comment. We have not included additional details regarding the specific diagnostic tests performed for the confirmation of CT, as providing a full description of all possible evaluations (including those not performed, such as PCR or IgA) would add substantial complexity to the manuscript and detract from the main objectives of our study. However, we can confirm that all CT cases were microbiologically confirmed within the REIV-TOXO network, according to the criteria defined in the Methods section (lines 202–205). We believe that these standardized criteria provide a robust and sufficient diagnostic framework for the purposes of our analysis.

**3. Conclusions**

**- Consider slightly reframing the concluding paragraph to emphasize how the evidence directly supports each claim (Reviewer #1)**

We thank the reviewer for this helpful suggestion. As recommended, we have revised the final conclusion to explicitly state that, based on the low IgM detection rate and the strong IgG concordance, DBS are suitable for seroepidemiologic surveillance but unsuitable for the diagnostic screening of CT. The concluding paragraph has been rewritten to integrate this statement while avoiding redundancies and ensuring coherence with the overall message of the manuscript (Lines 510-513)

We are grateful to all reviewers for their helpful comments, which have significantly improved the clarity and quality of our manuscript. We believe that the revised version fully addresses the concerns raised and strengthens the presentation and interpretation of our findings.

Sincerely,
Borja Guarch, on behalf of all authors
